# Supplementary material for: Ampelomyces strains isolated from diverse powdery mildew hosts in Japan: Their phylogeny and mycoparasitic activity, including timing and quantifying mycoparasitism of Pseudoidium neolycopersici on tomato
Source: PLoS One. 2021 May 11;16(5):e0251444. doi: 10.1371/journal.pone.0251444 (PMC8112701; doi:10.1371/journal.pone.0251444)
Supplement: Attachment — Submitted filename: PONE-D-20-39222_reviewer.pdf [file pone.0251444.s002.pdf]

PLOS ONE

Ampelomyces strains isolated from diverse powdery mildew hosts in Japan: Their phylogeny and mycoparasitic activity, including timing and quantifying mycoparasitism of Pseudoidium neolycopersici on tomato

--Manuscript Draft--

**Manuscript Number:**	PONE-D-20-39222
**Article Type:**	Research Article
**Full Title:**	Ampelomyces strains isolated from diverse powdery mildew hosts in Japan: Their phylogeny and mycoparasitic activity, including timing and quantifying mycoparasitism of Pseudoidium neolycopersici on tomato
**Short Title:**	Infection processes of Ampelomyces strains on/in powdery mildew fungi
**Corresponding Author:**	Teruo Nonomura<br>Laboratory of Phytoprotection, Science and Technology<br>NARA, JAPAN
**Keywords:**	conidiophore; hyperparasite; phylogenetic analysis; pycnidium formation; Solanum lycopersicum; tomato powdery mildew
**Abstract:**	A total of 26 Ampelomyces isolates were obtained from six naturally infected powdery mildew samples collected in Japan. We characterized the isolates based on morphological characteristics and sequences of ribosomal DNA internal transcribed spacer (rDNA-ITS) regions and actin (ACT) fragments. Japanese strains represented six different genotypes and were accommodated in three different clades of the genus Ampelomyces . Morphology of the strains agreed that of other Ampelomyces strains, but none of the examined characters was associated with any groups obtained in the genetic analysis. Mycoparasitic activity of selected Ampelomyces strains was determined by estimating the infectivity on five powdery mildew species. In these inoculation experiments, hyperparasites successfully infected all tested powdery mildew species, and showed no significant differences in their mycoparasitic activity as measured by the number of pycnidia developed in powdery mildews colonies during mycoparasitism. A detailed analysis on the mycoparasitic interaction between Ampelomyces and the tomato powdery mildew fungus (Pseudoidium neolycopersici KTP-03) revealed that the spores of the hyperparasite germinated on tomato leaves and its hyphae penetrated the KTP-03 hyphae. Ampelomyces hyphae continued their growth internally and caused its hosts conidiophores and hyphae to initiate atrophy at 5 days, to atrophy at 6 days and then to completely collapse at 7 days. Ampelomyces produced new intracellular pycnidia in KTP-03 conidiophores at ca. 8–10 days. Finally, KTP-03 hyphae were successfully destroyed by invasion of the hyperparasitic strain. Mature pycnidia released spores at ca. 10–14 days after inoculation, which became the sources of subsequent infections. Our study is first to report a detailed analysis on Japanese Ampelomyces strains, and on interactions between a hyperparasitic Ampelomyces strain and its mycohost using digital microscopic technologies.
**Order of Authors:**	Márk Z Németh
	Yuusaku Mizuno
	Hiroki Kobayashi
	Diána Seress
	Naruki Shishido
	Yutaka Kimura
	Susumu Takamatsu
	Tomoko Suzuki
	Yoshihiro Takikawa
	Yoshinori Matsuda
	Koji Kakutani

Powered by Editorial Manager® and ProduXion Manager® from Aries Systems Corporation

	Levente Kiss
	Teruo Nonomura
**Additional Information:**	
**Question**	**Response**
**Financial Disclosure**<br><br>Enter a financial disclosure statement that describes the sources of funding for the work included in this submission. Review the submission guidelines for detailed requirements. View published research articles from *PLOS ONE* for specific examples.<br><br>This statement is required for submission and **will appear in the published article** if the submission is accepted. Please make sure it is accurate.<br><br>**Unfunded studies**<br>Enter: *The author(s) received no specific funding for this work.*<br><br>**Funded studies**<br>Enter a statement with the following details:<br>• Initials of the authors who received each award<br>• Grant numbers awarded to each author<br>• The full name of each funder<br>• URL of each funder website<br>• Did the sponsors or funders play any role in the study design, data collection and analysis, decision to publish, or preparation of the manuscript?<br>• **NO** - Include this sentence at the end of your statement: *The funders had no role in study design, data collection and analysis, decision to publish, or preparation of the manuscript.*<br>• **YES** - Specify the role(s) played.<br><br>* typeset	No. The funders had no role in study design, data collection and analysis, decision to publish, or preparation of the manuscript.
**Competing Interests**<br><br>Use the instructions below to enter a competing interest statement for this submission. On behalf of all authors, disclose any competing interests that could be perceived to bias this work—acknowledging all financial support and any other relevant financial or non-	No. The authers have declared that no competing interests exist.

financial competing interests.<br><br>This statement **will appear in the published article** if the submission is accepted. Please make sure it is accurate. View published research articles from *PLOS ONE* for specific examples.<br><br>**NO authors have competing interests**<br><br>Enter: *The authors have declared that no competing interests exist.*<br><br>**Authors with competing interests**<br><br>Enter competing interest details beginning with this statement:<br><br>*I have read the journal's policy and the authors of this manuscript have the following competing interests: [insert competing interests here]*<br><br>* typeset	
**Ethics Statement**<br><br>Enter an ethics statement for this submission. This statement is required if the study involved:<br><br>• Human participants<br>• Human specimens or tissue<br>• Vertebrate animals or cephalopods<br>• Vertebrate embryos or tissues<br>• Field research<br><br>Write "N/A" if the submission does not require an ethics statement.<br><br>General guidance is provided below. Consult the submission guidelines for detailed instructions. **Make sure that all information entered here is included in the Methods section of the manuscript.**	This statement is not required in this study.

**Format for specific study types**<br><br>**Human Subject Research (involving human participants and/or tissue)**<br>• Give the name of the institutional review board or ethics committee that approved the study<br>• Include the approval number and/or a statement indicating approval of this research<br>• Indicate the form of consent obtained (written/oral) or the reason that consent was not obtained (e.g. the data were analyzed anonymously)<br><br>**Animal Research (involving vertebrate animals, embryos or tissues)**<br>• Provide the name of the Institutional Animal Care and Use Committee (IACUC) or other relevant ethics board that reviewed the study protocol, and indicate whether they approved this research or granted a formal waiver of ethical approval<br>• Include an approval number if one was obtained<br>• If the study involved *non-human primates*, add *additional details* about animal welfare and steps taken to ameliorate suffering<br>• If anesthesia, euthanasia, or any kind of animal sacrifice is part of the study, include briefly which substances and/or methods were applied<br><br>**Field Research**<br><br>Include the following details if this study involves the collection of plant, animal, or other materials from a natural setting:<br>• Field permit number<br>• Name of the institution or relevant body that granted permission	
**Data Availability**<br><br>Authors are required to make all data underlying the findings described fully available, without restriction, and from the time of publication. PLOS allows rare exceptions to address legal and ethical concerns. See the PLOS Data Policy and FAQ for detailed information.	Yes - all data are fully available without restriction

A Data Availability Statement describing where the data can be found is required at submission. Your answers to this question constitute the Data Availability Statement and **will be published in the article**, if accepted.<br><br>**Important:** Stating 'data available on request from the author' is not sufficient. If your data are only available upon request, select 'No' for the first question and explain your exceptional situation in the text box.<br><br>Do the authors confirm that all data underlying the findings described in their manuscript are fully available without restriction?	
**Describe where the data may be found in full sentences. If you are copying our sample text, replace any instances of XXX with the appropriate details.**<br><br>• If the data are **held or will be held in a public repository**, include URLs, accession numbers or DOIs. If this information will only be available after acceptance, indicate this by ticking the box below. For example: *All XXX files are available from the XXX database (accession number(s) XXX, XXX.)*.<br>• If the data are all contained **within the manuscript and/or Supporting Information files**, enter the following: *All relevant data are within the manuscript and its Supporting Information files.*<br>• If neither of these applies but you are able to provide **details of access elsewhere**, with or without limitations, please do so. For example:<br><br>*Data cannot be shared publicly because of [XXX]. Data are available from the XXX Institutional Data Access / Ethics Committee (contact via XXX) for researchers who meet the criteria for access to confidential data.*<br><br>*The data underlying the results presented in the study are available from (include the name of the third party*	All nucleotide sequences are available from the DNA Data Bank of Japan nucleotide sequence database (https://www.ddbj.nig.ac.jp/index-e.html) (accession numbers MT645844 to MT645849, MT644626 to MT644651 and MT656304 to MT656329). The other relevant data are within the paper and its Supporting Information files.

and contact information or URL).<br>• This text is appropriate if the data are owned by a third party and authors do not have permission to share the data.<br><br>* typeset	
Additional data availability information:	Tick here if the URLs/accession numbers/DOIs will be available only after acceptance of the manuscript for publication so that we can ensure their inclusion before publication.

Powered by Editorial Manager® and ProduXion Manager® from Aries Systems Corporation

Manuscript Click here to access/download;Manuscript;Manuscript 2.docx

Ampelomyces strains isolated from diverse powdery mildew hosts in Japan: Their phylogeny and mycoparasitic activity, including timing and quantifying mycoparasitism of *Pseudoidium neolycopersici* on tomato

Márk Z Németh[1¶] · Yuusaku Mizuno[2¶] · Hiroki Kobayashi[2] · Diána Seress[1] · Naruki Shishido[2] · Yutaka Kimura[2] · Susumu Takamatsu[3] · Tomoko Suzuki[4] · Yoshihiro Takikawa[5] · Koji Kakutani[6] · Yoshinori Matsuda[2] · Levente Kiss[1,#a] * · Teruo Nonomura[2,7]*

[1] Plant Protection Institute, Centre for Agricultural Research, Budapest, Hungary

[2] Laboratory of Phytoprotection, Science and Technology, Faculty of Agriculture, Kindai University, Nara, Japan

[3] Faculty of Bioresources, Mie University, Mie, Japan

[4] Department of Chemical Biological Sciences, Faculty of Science, Japan Women's University, Tokyo, Japan

[5] Plant Center, Institute of Advanced Technology, Kindai University, Wakayama, Japan

[6] Pharmaceutical Research and Technology Institute, Kindai University, Osaka, Japan

[7] Agricultural Technology and Innovation Research Institute, Kindai University, Nara, Japan

[#a] Centre for Crop Health, University of Queensland, Toowoomba, Australia

[¶]These authors contributed equally to this work.

* Corresponding author: Levente.Kiss@usq.edu.au (LK); nonomura@nara.kindai.ac.jp (TN)

Abstract

A total of 26 *Ampelomyces* isolates were obtained from six naturally infected powdery mildew samples collected in Japan. We characterized the isolates based on morphological characteristics and sequences of ribosomal DNA internal transcribed spacer (rDNA-ITS) regions and actin (*ACT*) fragments. ~~Japanese~~ strains represented six different genotypes and were accommodated in three different clades of the genus *Ampelomyces*. Morphology of the strains agreed that of other *Ampelomyces* strains, but none of the examined characters ~~was~~ associated with any groups obtained in the genetic analysis. Mycoparasitic activity of selected *Ampelomyces* strains was determined by estimating the infectivity on five powdery mildew species. In these inoculation experiments, hyperparasites successfully infected all tested powdery mildew species, and showed no significant differences in their mycoparasitic activity as measured by the number of pycnidia developed in powdery mildews colonies during mycoparasitism. A detailed analysis on the mycoparasitic interaction between *Ampelomyces* and the tomato powdery mildew fungus (*Pseudoidium neolycopersici* KTP-03) revealed that the spores of the hyperparasite germinated on tomato leaves and its hyphae penetrated the KTP-03 hyphae. *Ampelomyces* hyphae continued their growth internally and caused its hosts conidiophores and hyphae to initiate atrophy at 5 days, to atrophy at 6 days and then to completely collapse at 7 days. *Ampelomyces* produced new intracellular pycnidia in KTP-03 conidiophores at *ca.* 8–10 days. Finally, KTP-03 hyphae were successfully destroyed by invasion of the hyperparasitic strain. Mature pycnidia released spores at *ca.* 10–14 days after inoculation, which became the sources of subsequent infections. Our study is first to report a detailed analysis on Japanese *Ampelomyces* strains, and on interactions between a hyperparasitic *Ampelomyces* strain and its mycohost using digital microscopic technologies.

Keywords Conidiophore · Hyperparasite · Phylogenetic analysis · Pycnidium formation · *Solanum lycopersicum* · Tomato powdery mildew

Headings Infection processes of *Ampelomyces* strains on/in powdery mildew fungi

Introduction

Powdery mildew fungi (family *Erysiphaceae*), which are obligate biotrophic plant pathogens, cause leaf damage and reduce yields in host plants [1,2], therefore, cause losses in agriculture. To control powdery mildew, fungicides are regularly applied. However, frequent use of fungicides poses the risk of powdery mildew fungi becoming resistant [3]. In addition, there are other problems associated with fungicide use, such as possible negative side effects on plant physiology [4] or on biodiversity [5]. To mitigate these problems, there is an urgent need for developing new, alternative control strategies against powdery mildews. Physical or biological methods have been tested against powdery mildews on host plants to potentially complement or substitute chemical control [6]. For example, mycoparasites have been successfully used against powdery mildews as biocontrol agents (BCAs). Such BCAs include *Aphanocladium album* [7], *Pseudozyma flocculosa, Moesziomyces rugulosus* [8,9], *Gjaerumia minor* [10,11], *Lecanicillium lecanii* [9] and *Ampelomyces quisqualis* [12,13]. *Ampelomyces* spp. are slow-growing pycnidial fungi, which are widely distributed and well known hyperparasites of powdery mildew fungi on many cultivated and wild plants [14–16]. Several studies [17–19] have shown that

Ampelomyces strains belong to genetically distinct lineages. Although these linages are thought to represent distinct species of the genus *Ampelomyces* [20–22] and differences between colony morphology of strains belonging to distinct clades were also hinted [18], a taxonomic revision of the genus has not been performed yet [23].

Cross-inoculation experiments, including *in vitro* studies [21,24–26] and field experiments [23,27] involving different *Ampelomyces* strains and several powdery mildew fungal species have consequently shown that these mycoparasitic strains do not show strict host specificity, i.e. they are capable of infecting many host species irrespective of the original host. This lack of specificity [6] allows BCAs composed of a single strain to be applied against a wide range of powdery mildew species. Indeed, many works have shown the potential of *Ampelomyces* spp. as biocontrol agents against powdery mildews on various crops, such as *Erysiphe trifoliorum* on red clover [28], *Podosphaera leucotricha* on apple [29,30], *Podosphaera xanthii* on cucumber [18,24,30–36] and melon [37,38], *Erysiphe necator* on grapevine [14,18,39,40], *Blumeria graminis* on barley [41,42] and wheat [42], and several others [18,24,28,30,36,43–46]. Some *Ampelomyces* isolates have been developed as commercial biofungicide products such as AQ10® (Ecogen Incorporated, USA), Q-fect® (Green Biotech, Korea) and Powderycare® (AgriLife, India), and used against powdery mildews [16,22,47]. Efficacy of biocontrol, however, varied significantly between the experiments involving mycoparasitic tests with *Ampelomyces*, some reporting superior [48,49], others [31,50] suboptimal control. These contradictions might result from experimental differences, such as humidity [16] but might also reflect differences in the mycoparasitic activities of *Ampelomyces* strains belonging to distinct lineages [16,26],

and/or presence of physiological differences between genetically similar or uniform strains of *Ampelomyces* [18,23,26]. For the effective utilization of *Ampelomyces* strains for biocontrol purposes, detailed knowledge is needed not only on the host range, but also on other aspects of their biology. Improved visualization was considered as an ideal approach for such studies [51]. In a previous study aimed at a better visualization of the interaction between the plant and the tomato powdery mildew fungus we used the leaf type I trichomes (1.5–2.5 mm in length on a multicellular base with a small glandular tip, and abundant and longer than other types of trichomes; [52]) of common tomato (cv. Moneymaker) and powdery mildew development was followed on the trichomes using a high-fidelity digital microscope [53]. Thus, from the methodological point of view, trichomes were excellent sites for analyzing the interactions between plant cells and pathogens under natural environmental conditions. Based on this, we assumed that it is also possible to visualize the interactions among hyperparasites, mycohosts and plant cells (tritrophic relations) in more detail under the same conditions mentioned above.

Our main aims in the present study were 1) to reveal morphological and physiological characteristics and phylogenetic placement of *Ampelomyces* isolates originating from Japan, 2) to test and quantify the rate of mycoparasitism exerted against different host species and 3) to observe the infection process and pycnidiogenesis of *Ampelomyces* isolates in the tomato powdery mildew fungus in detail. To our knowledge, this is the first study to provide detailed analysis of Japanese *Ampelomyces* strains in terms of phylogeny, morphology, physiology and rate of mycoparasitism, and the first to consecutively monitor

the infection process of *Ampelomyces* isolates in its host using digital microscopic techniques.

Materials and methods

Identification of host powdery mildew fungi of the newly isolated *Ampelomyces* strains

Host powdery mildew fungi were identified based on morphology, host plants and ITS sequences. Morphological observation was done after preparing samples as in [54]. DNA extraction was conducted as described in [55]. For amplification of ITS in two steps (as detailed in [55], Phusion Green Hot Start II High-Fidelity PCR Master Mix (Thermo Fisher Scientific, MA, USA) was used with reaction mix composition and reaction conditions as recommended by the manufacturer and primer annealing temperature set to 58°C. Tubes lacking DNA template were included as negative controls. Amplicons resulting from PCRs were run on 1% agarose gel. Fragments were sent for sequencing to LGC Genomics GmbH (Germany). Sequencing was done with the same primers used for the amplifications. The resulting chromatograms were processed using Staden Program Package [56]. Sequences were deposited to Genbank under accession numbers MT645844–MT645849.

Isolation and culture of *Ampelomyces* strains

A total of 26 *Ampelomyces* strains were isolated from six naturally infected powdery mildew fungi samples collected in three Prefectures in Japan **(Table 1)**. Pycnidia were isolated from powdery mildew fungi as described in [21]. After isolation, colonies were transferred three times for purifying the colonies. Colonies of *Ampelomyces* strains were

cultured on Czapex-Dox agar medium supplemented with 2% malt extract (MCzA; 3g $NaNO_3$, 1g K_2HPO_4, 0.5g KCl, 0.5g $MgSO_4$, 15g agar and 20g malt extract) and maintained at 25 ± 2°C and continuous illumination of 22.2 $\mu moL \cdot m^{-2} \cdot s^{-1}$. Strains were subcultured every 2 months.

Table 1. Designations, hosts and isolation data and GenBank accession numbers of ITS and *ACT* sequences of the 26 *Ampelomyces* strains included in the current work.

Strain designations	Date of isolation	Place of collection	Host fungi	Host plants of powdery mildews	ITS accession numbers	*ACT* accession numbers
**Eq-h**	12 Oct 2017	Mie Prefecture, Japan	*Erysiphe quercicola*	*Quercus phillyraeoides*	MT644635	MT656313
Eq-k					MT644636	MT656314
**Eq-m**					MT644637	MT656315
**7100-a**	18 Oct 2017	Tochigi Prefecture, Japan	*Erysiphe glycines*	*Amphicarpaea bracteata*	MT644626	MT656304
**7100-b**					MT644627	MT656305
**7100-d**					MT644628	MT656306
7100-e					MT644629	MT656307
**7100-g**					MT644630	MT656308
**7124-e**	02 Nov 2017	Shiga Prefecture, Japan	*Erysiphe trifoliorum*	*Vicia* sp.	MT644631	MT656309
7134-b	02 Nov 2017	Shiga Prefecture, Japan	*Erysiphe orixae*	*Orixa japonica*	MT644632	MT656310
7134-d					MT644633	MT656311
7134-h					MT644634	MT656312
Xs-a	06 Nov 2017	Mie Prefecture, Japan	*Podosphaera xanthii*	*Xanthium stramonium*	MT644645	MT656323
Xs-c					MT644646	MT656324
Xs-h					MT644647	MT656325
Xs-m					MT644648	MT656326
Xs-s					MT644649	MT656327
**Xs-q**					MT644650	MT656328
Xs-y					MT644651	MT656329
Ga-a	05 Dec 2017	Mie Prefecture, Japan	*Golovinomyces asterum* var. *solidaginis*	*Solidago* sp.	MT644638	MT656316
Ga-b					MT644639	MT656317
Ga-c					MT644640	MT656318

Ga-d					MT644641	MT656319
Ga-e					MT644642	MT656320
Ga-f					MT644643	MT656321
Ga-g					MT644644	MT656322

DNA extraction from *Ampelomyces* strains, PCR amplification and sequencing

DNA was extracted from approximately 1 cm^2 of two-week-old *Ampelomyces* colonies using DNeasy Plant Mini Kit (Qiagen, MD, USA) according to the protocol provided with the kit. Following DNA extraction, two loci, ITS and partial *ACT* were amplified and sequenced. For amplification of ITS, primers ITS1F [57] and ITS4 [58] and the following PCR protocol were used: initial denaturation at 94°C for 5 min, followed by 35 cycles of 94°C for 45 s, 55°C for 45 s and 72°C for 1 min. Reactions ended with final incubation at 72°C for 10 min. Primers Act-1 and Act-5ra [59] were used for *ACT* amplifications with the following PCR protocol: initial denaturation at 98°C for 5 min, followed by 35 cycles of 98°C for 30 s, 54°C for 1 min and 72°C for 1 min. Reactions ended with final incubation at 72°C for 5 min. 20 µL reactions were run with DreamTaq Green PCR Master Mix (Thermo Fisher Scientific, MA, USA) using primers (Sigma-Aldrich, MO, USA) in 500 nM final concentration. PCR mixes lacking any DNA template were always included as negative controls. Sequencing and processing of chromatograms was conducted as detailed above. Sequences were deposited to Genbank under accession numbers MT644626–MT644651 (ITS) and MT656304–MT656329 (*ACT*).

Sequence alignments and phylogenetic analyses

Sequences obtained from the new Japanese isolates were analyzed together with ITS and *ACT* sequences of 103 other *Ampelomyces* strains determined earlier [17,22,27,51], and a *Phoma herbarum* strain (CBS 567.63) as an outgroup [17], thus the analysis included a total of 130 strains (including *Ph. herbarum*). Alignments and the final dataset to be analyzed were produced as described in [51]. The combined ITS_*ACT* dataset was analyzed with maximum likelihood (ML) and Bayesian inference (BI) methods with partitions set corresponding to ITS and *ACT*. ITS consisted of 501 characters and *ACT* consisted of 820 characters, thus the combined dataset included 1321 characters in total. For ML analysis raxmlGUI 1.5 [60,61] was used as detailed in [51]. For BI, MrBayes 3.1.2 [62] was used and the method described in [63] was followed. Majority (50%) rule consensus tree was calculated from the trees sampled in BI, omitting the first 4000 sampled trees (burn-in). Phylogenetic trees resulting from analyses were visualized in TreeGraph 2.14.0 [64] and submitted to TreeBASE (study ID 25861).

Morphological observations on *Ampelomyces in vitro* growth characteristics

Eight representative strains, originating from four independent powdery samples, and belonging to four distinct haplotypes (see Results) were selected for a more detailed analysis **(Table 2)**. These eight strains also included strains originating from the same powdery mildew sample, potentially representing strain-level differences in their characteristics. 30-day-old sporulating colonies of *Ampelomyces* strains were flushed with 1.0–1.5 mL of sterile distilled water. Colonies were scraped with a sterile scalpel to facilitate suspension of spores. The concentration of suspension was measured with a

haemocytometer (Nippon Rinsho Kikai Kogyo Co. Ltd., Tokyo, Japan) and it was diluted to 5 x 10^5 spores mL^{-1}. Tween 20 was added to a final concentration of 0.05%. Water agar plate overlaid with cellophane sheet was sprayed with the spores of *Ampelomyces* isolates and incubated at room temperature (25 ± 2°C). Spore germination rates and hyphal lengths were measured 3 days after inoculation under a KH-2700 high-fidelity digital microscope (KH-2700 DM; Hirox, Tokyo, Japan). Data are presented as means and standard deviations of five replicates (as 100 spores at one replication). Radial growth rate of *Ampelomyces* spp. colonies was evaluated by measuring the diameter of each colony after incubation for 20 days. Data are presented as means and standard deviations of five replicates (as 10 colonies at one replication).

Table 2 Morphological characteristics of *Ampelomyces* spp. fungi inoculated onto Czapex-Dox agar medium supplemented with 2% malt (MCzA).

Fungi tested	Spores				Germination rates (%) [a]		Hyphal lengths (μm) [b]		Colony areas (mm^2) [c]	
	Length (μm)		Width (μm)							
Eq-h	7.7 ± 1.5	a	4.0 ± 1.0	a	79.3 ± 9.0	a	44.5 ± 21.6	a	271.6 ± 119.7	a
Eq-m	6.6 ± 0.8	a	3.2 ± 0.5	a	74.2 ± 8.6	ab	29.7 ± 18.5	b	211.1 ± 46.3	a
Xs-q	7.0 ± 1.1	a	3.2 ± 0.6	a	80.0 ± 8.8	a	29.6 ± 18.1	b	263.1 ± 68.6	a
7100-a	6.5 ± 0.8	a	3.0 ± 0.4	a	72.7 ± 8.0	ab	28.7 ± 16.9	bc	247.2 ± 79.8	a
7100-b	6.6 ± 0.8	a	3.2 ± 0.5	a	81.5 ± 10.4	a	47.3 ± 21.9	a	274.8 ± 64.9	a
7100-d	6.8 ± 0.7	a	3.3 ± 0.4	a	93.0 ± 2.2	a	55.6 ± 22.6	d	236.6 ± 88.1	a
7100-g	7.3 ± 0.8	a	3.3 ± 0.5	a	69.1 ± 5.5	b	35.5 ± 21.1	bc	227.2 ± 118.2	a
7124-e	7.2 ± 0.8	a	3.6 ± 0.4	a	94.2 ± 3.9	a	34.4 ± 14.5	b	228.7 ± 77.3	a

[a] Germination of *Ampelomyces* spp. spores was observed 24 h after inoculation.

[b] Hyphal lengths of *Ampelomyces* spp. fungi were measured 48 h after inoculation.

[c] Colony areas of *Ampelomyces* spp. fungi were measured 20 days after inoculation of single pycnidia onto center of MCzA medium.

Different letters in each column indicate significant difference ($p < 0.05$, Tukey's method).

Plant material

Seeds of the common tomato *Solanum lycopersicum* Mill. cv. Moneymaker (MM), obtained from self-pollinated progenies in our greenhouse, were germinated on water-soaked filter papers in a Petri dish for 3 days in an LH-240N growth chamber (Nippon Medical & Chemical Instruments, Osaka, Japan) under continuous illumination (19.8–40.3 $\mu moL \cdot m^{-2} \cdot s^{-1}$; 400–700 nm) with white (full-spectrum) fluorescent lamps FL40SS W/37 (Mitsubishi, Tokyo, Japan) at 25 ± 2°C. Seedlings at the cotyledon stage were placed into polyurethane cubic sponge supports (3 x 3 x 3 cm^3). The sponge supports with seedlings were inserted into the 30 mL cylindrical plastic case (diameter 3 cm, length 5 cm) containing 20 mL hydroponic nutrient solution (4.0 mM KNO_3, 1.5 mM $Ca(NO_3)_2$, 1.0 mM $MgSO_4$, 0.66 mM $NH_4H_2PO_4$, 0.057 mM FeEDTA, 0.048 mM H_3BO_3 and 0.009 mM $MnSO_4$) and then incubated for 14 days in a temperature-controlled room under the following conditions: 25 ± 2°C, 50–70% relative humidity (RH) and continuous illumination of 59.5 $\mu moL \cdot m^{-2} \cdot s^{-1}$.

Powdery mildew material, inoculation, and incubation

A tomato powdery mildew isolate (*Pseudoidium neolycopersici* L. Kiss KTP-03, [65]), continuously maintained in the growth chamber was used in this study. Mature conidia

were collected from the fungal colonies on infected leaves using an electrostatic spore collector as described previously [66] and transferred onto the true leaves of 14-day-old tomato healthy seedlings (MM) under a KH-2700 DM. The inoculated seedlings were incubated for 40 days in growth chambers at 25 ± 1°C and 50–70% RH under continuous illumination of 22.2 µmoL·m^{-2}·s^{-1} [65].

Mycoparasitic tests with Japanese *Ampelomyces* strains

The same eight *Ampelomyces* strains as in the morphological characterization were included in the mycoparasitic tests. The following powdery mildew species maintained in the greenhouse were used: *Blumeria graminis* f. sp. *hordei* Marchal race 1 KBP-01 (on barley), *Erysiphe trifoliorum* Greville KRCP-4N (on red clover), *Podosphaera aphanis* U. Braun & S. Takamatsu KSP-7N (on strawberry), *Podosphaera xanthii* Pollacci KMP-6N (on melon) and *Pseudoidium neolycopersici* L. Kiss KTP-03 (on tomato). New powdery mildew colonies used in the experiments were 10 days old. *Ampelomyces* spore suspension was prepared as above. Five powdery mildew infected plants for each strain were inoculated with the *Ampelomyces* spore suspension, and placed in plastic boxes. Five uninoculated plants for each strain served as controls. Gauze pads were put in the boxes and wetted with sterile tap water. The boxes were closed and incubated for 10–20 days in growth chambers at 25 ± 1°C and 80–90% RH under continuous illumination of 22.2 µmoL·m^{-2}·s^{-1}.

Morphological analysis of pycnidia and conidia developed during mycoparasitism

Ampelomyces pycnidia and conidia developed during mycoparasitic tests were observed with an SZ60 stereomicroscope (SZ60 SM; Olympus, Tokyo, Japan) and a KH-2700 DM. Length and width of mature pycnidia and spores formed in tomato powdery mildew colonies grown for 14 days at 25 ± 2°C in the dark were measured on glass slides under a KH-2700 DM. Data are presented as means and standard deviations of five replicates (as 20 pycnidia and 100 spores at one replication).

Quantification of mycoparasitic activity

To evaluate the infectivity of the *Ampelomyces* spp. strains, the number of pycnidia formed in ten powdery mildew colonies of the five powdery mildew fungal species listed above was counted 14 days after inoculation. Number of pycnidia was scored on a three level scale of: no pycnidia present, between 11 and 100 pycnidia, and 101 or more pycnidia formed on single powdery mildew colony. In addition, the number of *Ampelomyces* spores produced in pycnidia developed in tomato powdery mildew was counted under a KH-2700 DM. Twenty leaf segments (approximately 1 x 1 cm^2 in size) were taken from ten powdery mildew-inoculated tomato plants. The samples were directly observed under the KH-2700 DM. Data are presented as means and standard deviations of five replicates (as 5 pycnidia at one replication).

Analysis of the mycoparasitic process of *Ampelomyces* strains in the tomato powdery mildew fungus *Ps. neolycopersici*

Isolate Xs-q was selected for a more detailed analysis on the mycoparasitic process of *Ampelomyces* strains into powdery mildew structures. To gain a general overview on the process of mycoparasitism and to observe the development of pycnidia in detail, Xs-q was spray-inoculated onto powdery-mildew infected tomato plants as detailed above. Inoculated leaves were observed regularly using the KH-2700 DM. In addition, samples were prepared for a BX-60 light microscope (BX-60 LM; Olympus, Tokyo, Japan). For this, samples were fixed and chlorophyll was removed in a boiling alcoholic lactophenol solution (10 mL glycerol, 10 mL phenol, 10 mL lactic acid, 10 mL distilled water and 40 mL 99.8% ethanol) for 1–2 min and then stained with 0.1% Aniline Blue (Nacalai Tesque, Tokyo, Japan) dissolved in distilled water as described previously [67]. The samples were observed under a BX-60 LM.

To reveal more details on the mycoparasitism, and in particular also for the direct observation of the infection process of *Ampelomyces* strains in real-time, the following experiment was designed. Mature conidia of *Ps. neolycopersici* KTP-03 were inoculated directly onto type I trichomes of 14-day-old MM plants [66] and the inoculated plants were incubated in the growth chamber for 10–14 days under the same conditions as described above. Powdery mildew grown on trichomes provided natural environmental conditions which are also well suited for observations [53]. The infection process of *Ampelomyces* strains in KTP-03 was observed using the KH-2700 DM. For this, single mature pycnidia of the *Ampelomyces* strain Xs-q grown on MCzA plates were placed onto *Ps. neolycopersici* KTP-03 colonies using glass needle micromanipulator [68]. The pycnidia served as a source of conidia, initiating the infection process to be observed. Hyphal development of

the strain Xs-q was photographed during 14 days after inoculation, using the 1/2’’ Interline transfer charge-coupled device (CCD) camera of the KH-2700 DM. The micrographs were analyzed using Adobe Photoshop image-processing software (ver. 5.0; Adobe Systems, CA, USA) for better contrasting of the captured images without changing the original information.

Results

Sequence analysis of ITS and *ACT* regions of *Ampelomyces* isolates

ITS and a region of *ACT* gene were determined from a total of 26 newly isolated *Ampelomyces* strains originating from Japan **(Table 1)**. Sequences of *Ampelomyces* strains isolated from the same powdery mildew sample were identical. Strains from the six independent powdery mildew samples represented five distinct ITS genotypes, as strains isolated from *Erysiphe quercicola* and *E. glycines* had identical ITS sequences. *ACT* sequences revealed six distinct genotypes.

Analysis of phylogeny of *Ampelomyces* isolates

ML and BI analyses on the combined ITS_*ACT* dataset revealed very similar phylogenies and identical groupings of clades. The tree with the highest likelihood is shown in **Fig 1**. Phylogenetic analysis of *Ampelomyces* strains revealed five distinct, well supported (bootstrap supports ≥ 94 percent; posterior probabilities = 1) clades. The largest clade, designated clade 1, consisted of 60 strains, including eight *Ampelomyces* strains isolated in this work. This clade was revealed to be phylogenetically diverse, comprising of several

subgroups. The eight Japanese strains clustered in clade 1 belonged to two distinct genotypes. Clade 2 contained 13, and clade 3 contained 15 strains, the latter including seven newly isolated strains. The second-largest clade was composed of 22 strains, half of which were isolated in this work. Thus, most of the strains isolated in this work belong to this clade, incorporating three different genotypes of Japanese isolates. Lastly, clade 5 contained 19 strains, none of which was isolated in Japan. Consequently, strains isolated from six independent powdery mildew samples in Japan were accommodated in three major distinct genetic clades of *Ampelomyces*.

Morphological observation of Japanese *Ampelomyces* isolates grown *in vitro*

Morphological characteristics were observed at detail in eight selected isolates. First our focus was on spore sizes and shapes, germination rates, initial times of germination, hyphal lengths and colony areas. Of eight *Ampelomyces* isolates in this study, the spores with unicellular, hyaline and ellipsoid-ovoid to doliiform morphology were *ca*. 5.7–9.2 x 2.6–5.0 µm. Spores germinated *ca*. 15–20 h after inoculation, elongated hyphae and then branched under conditions of high RH. The lengths of all hyphae formed from spores were *ca*. 6.2–78.2 µm at 48 h after inoculation. Moreover, fungal colonies slowly and concentrically spread after inoculation of a single mature pycnidium on the center of MCzA media. The colony areas were *ca*. 148.4–391.3 mm^2 at 20 days after inoculation. There were significant differences in germination rates and hyphal lengths among the isolates, but not in colony areas (**Table 2**).

Infectivity tests of Japanese *Ampelomyces* strains to powdery mildew fungi

To evaluate their infectivity, eight Japanese *Ampelomyces* strains were individually inoculated onto five different powdery mildew fungi maintained in our laboratory. In the tests, hyperparasites successfully infected all five powdery mildew isolates and formed mature pycnidia in four out of five mycohost colonies (melon, red clover, strawberry and tomato powdery mildew fungi), but not in barley powdery mildew. The tested strains infected the melon powdery mildew KMP-6N more heavily than others, as reflected by forming more pycnidia than in the other tested powdery mildew isolates at 14 days after inoculation onto powdery mildew colonies. There were no significant differences in mycoparasitic activity of the eight *Ampelomyces* strains based on the three-level scoring. **Table 3** shows the data obtained in the cross-inoculation experiments.

Table 3 Mycoparasitic tests of Ampelomyces spp. fungi to powdery mildew fungi (PM) and morphological characteristics of pycnidia produced in conidiophores of tomato powdery mildew KTP-03.

Fungi tested	Formation of mature pycnidia in/on hyphae of powdery mildew fungi					Pycnidia				Numbers of spores produced in a mature pycnidium [c]	
	Barley PM [a]	Melon PM	Red clover PM	Strawberry PM	Tomato PM	Length (μm)		Width (μm)			
Eq-h	− [b]	++	+	+	+	72.4 ± 11.8	a	40.8 ± 7.3	a	426.3 ± 222.8	bc
Eq-m	−	++	+	+	+	57.0 ± 15.6	ab	31.0 ± 7.7	ab	552.8 ± 206.1	b
Xs-q	−	++	+	+	+	55.1 ± 14.8	b	38.9 ± 7.6	a	493.2 ± 112.6	b
7100-a	−	++	+	+	+	63.7 ± 11.3	a	35.3 ± 6.0	a	1064.9 ± 427.8	a
7100-b	−	++	+	+	+	59.6 ± 16.3	ab	32.7 ± 6.4	ab	1025.0 ± 274.2	a
7100-d	−	++	+	+	+	67.0 ± 11.5	a	36.5 ± 6.2	a	590.8 ± 183.3	bc
7100-g	−	++	+	+	+	62.0 ± 16.6	ab	29.3 ± 6.7	b	317.2 ± 117.8	c
7124-e	−	++	+	+	+	53.6 ± 13.4	b	30.8 ± 7.6	ab	569.0 ± 255.7	b

[a] 10 ten-day-old powdery mildew colonies were used in this experiment.

[b] To evaluate the infectivity of the *Ampelomyces* spp. to powdery mildew fungi, the numbers of pycnidia formed on powdery mildew colonies were counted 14 days after spray inoculation of spores onto single powdery mildew colonies. −, +, and ++ represent no pycinidia, between 11 and 100 pycnidia, and more than 101 pycnidia formed on single powdery mildew colony, respectively.

[c] The numbers of spores in newly formed pycnidium was counted 14 days after spray inoculation of spores onto 10-day-old tomato powdery mildew KTP-03 mycelia.

Different letters in each column indicate significant difference ($p < 0.05$, Tukey's method).

Morphology of pycnidia developed in tomato powdery mildew colonies and quantification of mycoparasitism

Sizes and shapes of the newly developed pycnidia and the number of spores produced in a pycnidium in tomato powdery mildew colonies were observed in detail and data are shown in **Table 3**. Pycnidia started to form *ca*. 6–7 days after spray inoculation of *Ampelomyces* spores onto 10-day-old KTP-03 mycelia **(Fig 2A)**. **Fig 2A** shows the mature pycnidia (Py1) with black-color and ovoid in shape, and immature pycnidia with pale yellows (Py2). The pycnidia were mainly produced in conidiophores of mycohosts **(Fig 2B)**. Mature pycnidia had sizes of *ca*. 40.2–84.2 x 22.6–48.1 μm, and the numbers of spores produced in a single mature pycnidium were *ca*. 199.4–1492.7 spores at 14 days after spray inoculation of *Ampelomyces* spores. As shown in **Fig 2C**, numerous spores were released from intracellular pycnidia by the rupture of the pycnidial wall after a drop of distilled water (10

μm). There were significant differences among the strains in the number of spores developed in a single pycnidium, and also the sizes of pycnidia **(Table 3)**.

Infection process of *Ampelomyces* isolate Xs-q in *Ps. neolycopersici*

Ps. neolycopersici KTP-03 formed lobed primary appressoria, elongated colony-forming hyphae from the conidial bodies within 48 h after inoculation of conidia onto the type I trichome cells, and successfully infected the trichomes. KTP-03 hyphae growing from the trichomes developed vigorously on the epidermal cells. KTP-03 formed conidiophores within 7 days after attachment of the tip of hyphae from the trichome cells on the epidermal cells **(Fig 3A)**. After spray inoculation of Xs-q spores, the spores germinated on tomato leaves and penetrated the KTP-03 conidia **(Fig 3B)** or/and hyphae, and continued their growth internally from cell to cell of KTP-03 hyphae **(Fig 3C)**. Eventually, Xs-q produced new intracellular pycnidia in KTP-03 conidiophores at *ca.* 8–10 days after inoculation **(Fig 3D)** and released spores from mature pycnidia at *ca.* 10–14 days after inoculation **(Fig 3E)**. The mature spores, which were released from intracellular pycnidia by the rupture of the pycnidial cell walls, became the sources of subsequent infection to the powdery mildew host fungus. Finally, KTP-03 hyphae were destroyed by invasion of the hyperparasite **(Fig 3F)**.

During mycoparasitism, we focused on the invasion into host structures, and the development of pycnidia in particular. We consecutively observed the invasion process of isolate Xs-q in KTP-03 hyphae using a KH-2700 DM, taking advantage of KTP-03-infected type I trichome cells. After inoculation of single Xs-q pycnidia onto tomato leaf in

close vicinity to the trichomes **(Fig 4A)**, the Xs-q spores released from the pycnidia germinated, developed hyphae which elongated towards KTP-03 hyphae **(Fig 4B and C)**. By invasion of Xs-q hyphae into KTP-03 hyphae, KTP-03 conidiophores and hyphae initiated atrophy at 5 days **(Fig 4D)** and atrophied at 6 days **(Fig 4E)** and then completely collapsed at 7 days after inoculation **(Fig 4F)**. In addition, the results showed that Xs-q hyphae that invaded KTP-03 conidiophores grew out from conidia formed at top of the conidiophores **(Fig 4D–F)**.

For the detailed observations on pycnidial development, tomato powdery mildew colonies infected with *Ampelomyces* strains following spray inoculation were used. **Fig 5A** shows well developed KTP-03 conidiophores. Foot and generative cells of KTP-03 conidiophores initiated to atrophy at *ca*. 5–6 days after spray inoculation of Xs-q onto KTP-03 colonies **(Fig 5B)**. In addition, Xs-q hyphae that invaded KTP-03 conidiophores, grew out from conidia formed at top of the conidiophores. Intracellular pycnidia of Xs-q initiated to produce in basal cells of the conidiophores at *ca*. 6–8 days after the spray inoculation **(Fig 5C)**. Moreover, Xs-q hyphae and pycnidia continued to elongate and grow in KTP-03 hyphae, respectively **(Fig 5D)**. Xs-q pycnidia changed immature (pale yellow) to mature (black color). Consequently, the conidiophores completely collapsed at *ca*. 10–14 days after the spray inoculation **(Fig 5E)**. Abundant spores were released from the mature pycnidia after treatment of a drop of distilled water (20 µL). The released mature spores became the sources of subsequent infections to KTP-03 hyphae (data not shown).

Discussion

In this study, we characterized *Ampelomyces* strains, potential BCAs [16] isolated from powdery mildew species in Japan, based on morphology and molecular phylogenetic analyses. To the best of our knowledge, this is first study to report such detailed analysis on *Ampelomyces* strains isolated in Japan. The morphological characteristics of Japanese isolates were ~~the~~ similar to those described by other works [69,70]. It was reported that the slow-growing and fast-growing strains belong to two distinct groups based on a molecular phylogenetic analysis of rDNA-ITS sequences [71]. The slow-growing strains are genetically more diverse and represent *Ampelomyces*, whereas fast-growing ones show a close phylogenetic relationship with *Epicoccum nigrum*, which is known to have a pycnidial, *Phoma*-like state [6]. In fact, ~~it seemed that~~ our isolates always ~~grow~~ slowly with an *in vitro* radial growth rate of 0.5–1.0 mm·d^{-1} on MCzA and were close to fungal developments of other *Ampelomyces* isolates [41,71].

Many molecular analyses based on the ITS region of the nuclear ribosomal RNA gene (nrDNA), and also *ACT* sequences have revealed considerable genetic diversity among *Ampelomyces* strains [17,19,21,22,25,51]. Using sequences from two loci, we confirmed the existence of at least five different genetic clades within *Ampelomyces* spp. reported earlier and showed that the newly isolated strains belong to three major clades. We analyzed phenotypic characteristics of *Ampelomyces* strains isolated from four different powdery mildew samples, and also four different strains isolated from the same powdery mildew sample. In our results, we did not detect morphological characteristics which could clearly be associated with given genotypes or clades. Four strains isolated from the same powdery mildew sample, however, displayed significant differences in measured hyphal

lengths, germination rates and number of spores developed in a pycnidium. These undoubtedly show the presence of differences on strain level, a phenomenon also observed in other works [23,26]. We do not know if the differences in the phenotypic characteristics of different strains of *Ampelomyces* are somehow related to their yet unrevealed genetic diversity or are simply caused by phenotypic plasticity. The possibility of strain-level differences, however, needs to be taken in consideration in upcoming studies, such as during development of potential BCAs.

We conducted tests using the spore suspensions of the hyperparasitic *Ampelomyces* strains. The concentration of hyperparasite spores is an important factor affecting their germination and infection. Germination of *Ampelomyces* spores decreases rapidly when the spores are at a concentration of more than 10^6 spores·mL^{-1}, due to the production of self-inhibitory substances [72]. Therefore, ~~in~~ this study~~,~~ used a lower concentration of spores (5 x 10^5 spores·mL^{-1}) for spray inoculation ~~to~~ nutrient media and powdery mildew-infected leaves. Consequently, Japanese isolates of *Ampelomyces* successfully germinated at 10–20 h after spray inoculation of the spores onto MCzA and powdery mildew-infected plants under conditions of high RH.

Earlier cross-inoculation experiments have shown that *Ampelomyces* hyperparasites collected from a given powdery mildew species can produce intracellular pycnidia in mycelia of other species of the Erysiphaceae [24,73,74]. After the cross inoculations with eight Japanese isolates into five powdery mildew species, we could observe the degeneration and constriction of hyphae of all five powdery mildew fungi tested, and also pycnidial formation in the hyphae and conidiophores of four powdery mildew fungi. The

experimental results of the cross-inoculation showed that Japanese *Ampelomyces* isolates infected powdery mildew hyphae irrespective of the original host and produced intracellular pycnidia in mycelia of tested species of the Erysiphaceae family. This is in accordance with several other studies [25–27,39,73] reporting lack of host specificity with the tested *Ampelomyces* isolates. Concerning *Ampelomyces* infectivity on *B. graminis*, it was observed that a strain isolated from *Erysiphe artemisiae* showed no infectivity to powdery mildew fungus (*Blumeria graminis* Speer) on barley (*Hordeum vulgare* L.) [69]. On the other hand, ~~an other~~ study showed hyphae advancing along *B. graminis* conidiophore on cereals (wheat and barley) and also spherical *Ampelomyces* pycnidia developing in the conidiophores [42]. Thus, the results of cross-inoculation experiments for barley powdery mildew fungi have been inconsistent over the range of hyperparasites tested. These contradictory results and the weak mycoparasitism exerted by the Japanese strains on *B. graminis* in our experiments might have been caused by unfavorable experimental conditions [16] as strains originating from non-*Blumeria* hosts were in most cases capable of infecting *B. graminis* [24,29].

In an earlier study [53], it was demonstrated that the tomato trichomes provide an interaction site that can be used as infection sites of powdery mildew fungi. ~~In the current study, therefore, w~~e took advantage of trichome cells projecting from host plant leaves and extended the use of the method for the detailed morphological analysis of hyperparasite-mycohost interactions~~. We were interested in~~ determining how and when the hyperparasites invade powdery mildew structures. ~~Based on earlier works and on our results obtained with detailed microscopic analysis, the course of infection and its approximate temporal process~~

can be summarized as follows. *Ampelomyces* might be transported for long distances by wind within the parasitized powdery mildew conidia [34,75,76] and spores in pycnidia, wet by rain, can be dispersed by rain splash [77,78]. Consequently, this is the way how these hyperparasites spread to the next infection source. It was reported that germination of *Ampelomyces* is significantly enhanced in the presence of conidia of powdery mildew fungi [34]. Moreover, a water-soluble substance secreted from powdery mildew conidia was shown to activate the spore germination of *A. quisqualis in vitro* [72]. These hint that the hyperparasite somehow recognizes the presence of mycohosts. The presence of the mycohost, however, is not necessary for germination as a study [51] showed that *Ampelomyces* also germinates in powdery mildew-free environments. These results also revealed that *Ampelomyces* strains might persist on the leaves without an immediate contact with their host, up to three weeks. When the host is present, *Ampelomyces* is able to penetrate and parasitize hyphae of powdery mildew fungi through mechanical [77] or enzymatic processes [26,79] after germination. Some works described that penetration of hyperparasites into mycohost structures can occur within 24 h [38,77]. Then, the hyphae of hyperparasites continue their growth in powdery mildew structures. The internal growth from cell to cell happens through the septal pores [77,80]. The invasion of *Ampelomyces* hyphae into the mycohost leads to the atrophy (in 5–6 days) and then to complete disruption of the mycohost conidiophores 7 days after inoculation. Not long after the infection occurs, the hyperparasites invade, ramify throughout the hyphae of mycohosts and destroy cytoplasm of their fungal hosts, resulting in reduced growth, and eventually death, of the host fungus [14,39,80]. In the meantime, *Ampelomyces* produces intracellular

pycnidia in the mycelia or within conidiophores of mycohosts at 5 to 10 days [32,74,77] after penetration of its hyphae into powdery mildew hyphae. Based on our digital microscopic observation, pycnidia initiated in 6–8 days and were morphologically mature by 8–10 days after inoculation. Almost all pycnidia were produced in conidiophores of powdery mildew fungi (see **Fig 3**). Eventually, spores are released from intracellular pycnidia by the rupture of both the pycnidial and the powdery mildew cell walls, which in our experiments happened at *ca*. 10–14 days after inoculation.

To our knowledge, the whole developmental process of *Ampelomyces* strains in powdery mildew fungi with an accompanying detailed microscopic analysis has not been presented yet. In this study, we consecutively demonstrated the developmental process of an *Ampelomyces* strain in a powdery mildew fungus using light and digital microscopes. The data obtained in the present study describing the interactions between *Ampelomyces* hyperparasites and mycohosts provide important base for upcoming studies on improving practical strategies to control powdery mildews. For example, *Ampelomyces* has been reported to tolerate a number of fungicides, acaricides and insecticides [34,35,38,74]. As next steps, we will examine the tolerance of Japanese *Ampelomyces* isolates to commercial fungicides and insecticides and focus on developing potent biological agents against powdery mildews.

Data Availability Statement: All nucleotide sequences are available from the DNA Data Bank of Japan nucleotide sequence database (https://www.ddbj.nig.ac.jp/index-e.html) (accession numbers MT645844 to MT645849, MT644626 to MT644651 and MT656304 to MT656329). The other relevant

data are within the paper and its Supporting Information files.

Funding: This work was partly supported by Grants for Scientific Research from Faculty of Agriculture, Kindai University, and Agricultural Technology and Innovation Research Institute, Kindai University. MZ Németh's stay in Japan was supported by the Institute of Fermentation, Osaka. The funders had no role in study design, data collection and analysis, decision to publish, or preparation of the manuscript.

Competing interests: The authors have declared that no competing interests exist.

Author Contributions

Conceptualization: L Kiss, T Nonomura.

Data curation: MZ Németh, Y Mizuno, L Kiss, T Nonomura.

Formal analysis: MZ Németh, Y Mizuno, S Takamatsu, Y Matsuda.

Funding acquisition: MZ Németh, S Takamatsu, T Nonomura.

Investigation: MZ Németh, Y Mizuno, H Kobayashi, D Seress, N Shishido, Y Kimura, T Suzuki, Y Takikawa, K Kakutani.

Methodology: MZ Németh, Y Mizuno, D Seress, Y Matsuda.

Project administration: MZ Németh, L Kiss, T Nonomura.

Resources: MZ Németh, L Kiss, S Takamatsu, T Nonomura.

Supervision: L Kiss, S Takamatsu, T Nonomura.

Visualization: MZ Németh, L Kiss, T Nonomura.

Writing – original draft preparation: MZ Németh, Y Mizuno.

Writing – review & editing: L Kiss, T Nonomura.

References

1. Matsuda Y, Kashimoto K, Takikawa Y, Aikami R, Nonomura T, Toyoda H. Occurrence of new powdery mildew on greenhouse tomato cultivars. J Gen Plant Pathol. 2001; 67:294–298.
2. White Jr JF, Johnston SA, Wang C-L, Chin C-K. First report of powdery mildew in greenhouse-grown tomatoes in New Jersey. Plant Dis. 1997; 81:227.
3. Rallos LEE, Baudoin AB. Co-occurrence of two allelic variants of CYP51 in *Erysiphe necator* and their correlation with over-expression for DMI resistance. PLoS One 2016; 11:e0148025.
4. Petit AN, Fontaine F, Vatsa P, Clément C, Vaillant-Gaveau N. Fungicide impacts on photosynthesis in crop plants. Photosyn Res. 2012; 111:315–326.
5. Geiger F, Bengtsson J, Berendse F, Weisser WW, Emmerson M, Morales MB, et al. Persistent negative effects of pesticides on biodiversity and biological control potential on European farmland. Basic Appl Ecol. 2010; 11:97–105.
6. Kiss L, Russell JC, Szentiványi O, Xu X, Jeffries P. Biology and biocontrol potential of *Ampelomyces mycoparasites*, natural antagonists of powdery mildew fungi. Biocont Sci Technol. 2004; 14:635–651.
7. Hijwegen T, Buchenauer H. Isolation and identification of hyperparasitic fungi associated with Erysiphaceae. Neth J Plant Pathol. 1984; 90:79–84.
8. Jarvis WR, Shaw LA, Traquair JA. Factors affecting antagonism of cucumber powdery mildew by *Stephanoascus flocculosus* and *S. rugulosus*. Mycol Res. 1989; 92:162–165.

9. Verhaar MA, Hijwegen T, Zadoks JC. Glasshouse experiments on biocontrol of cucumber powdery mildew (*Sphaerotheca fuliginea*) by the mycoparasites *Verticillium lecanii* and *Sporothrix rugulosa*. Biol Control 1996; 6:353–360.

10. Hoch HC, Provvidenti R. Mycoparasitic relationships: cytology of the *Sphaerotheca fuliginea*-*Tilletiopsis* sp. interaction. Phytopathology 1979; 69:359–362.

11. Hijwegen T. Biological control of cucumber powdery mildew by *Tilletiopsis minor*. Neth J Plant Pathol. 1986; 92:93–95.

12. Cesati V. *Ampelomyces quisqualis* Ces. Botanische Zeitung. 1852; 10:301–302. (also in Flora, 1852; 35:395–399)

13. Yarwood CE. An overwintering pycnidial stage of *Cicinnobolus*. Mycologia 1939; 31:420–422.

14. Falk SP, Gadoury DM, Cortesi P, Pearson RC, Seem RC. Parasitism of *Uncinula necator* cleistothecia by the mycoparasite *Ampelomyces quisqualis*. Phytopathology 1995a; 85:794–800.

15. Kiss L. Natural occurrence of *Ampelomyces* intracellular mycoparasites in mycelia of powdery mildew fungi. New Phytol. 1998; 140:709–714.

16. Kiss L. A review of fungal antagonists of powdery mildews and their potential as biocontrol agents. Pest Manag Sci. 2003; 59:475–483.

17. Pintye A, Bereczky Z, Kovács GM, Nagy LG, Xu X, Legler SE, et al. No indication of strict host associations in a widespread mycoparasite: grapevine powdery mildew (*Erysiphe necator*) is attacked by phylogenetically distant *Ampelomyces* strains in the field. Phytopathology 2012; 102:707–716.

18. Angeli D, Maurhofer M, Gessler C, Pertot I. Existence of different physiological forms within genetically diverse strains of *Ampelomyces quisqualis*. 2012a; Phytoparasitica 40:37–51.

19. Liyanage KK, Khan S, Brooks S, Mortimer PE, Karunarathna SC, Xu J, et al. Morpho-molecular characterization of two *Ampelomyces* spp. (Pleosporales) strains mycoparasites of powdery mildew of *Hevea brasiliensis*. Front Microbiol. 2018; 9:1–10.

20. Kiss L. Genetic diversity in *Ampelomyces* isolates, hyperparasites of powdery mildew fungi, inferred from RFLP analysis of the rDNA ITS region. Mycol Res. 1997b; 101:1073–1080.

21. Liang C, Yang J, Kovács GM, Szentiványi O, Li B, Xu X, et al. Genetic diversity of *Ampelomyces* mycoparasites isolated from different powdery mildew species in China inferred from analyses of rDNA ITS sequences. Fungal Diversity 2007; 24:225–240.

22. Park M-J, Choi Y-J, Hong S-B, Shin H-D. Genetic variability and mycohost association of *Ampelomyces quisqualis* isolates inferred from phylogenetic analyses of ITS rDNA and actin gene sequences. Fungal Biol. 2010; 114:235–247.

23. Legler SE, Pintye A, Caffi T, Gulyás Sz, Bohár Gy, Rossi V, et al. Sporulation rate in culture and mycoparasitic activity, but not mycohost specificity, are the key factors for selecting *Ampelomyces* strains for biocontrol of grapevine powdery mildew (*Erysiphe necator*). Eur J Plant Pathol. 2016; 144:723–736.

24. Sztejnberg A, Galper S, Mazar S, Lisker N. *Ampelomyces quisqualis* for biological and integrated control of powdery mildews in Israel. J Phytopathol. 1989; 124:285–295.

25. Szentiványi O, Kiss L, Russell JC, Kovács GM, Varga K, Jankovics T, et al. *Ampelomyces* mycoparasites from apple powdery mildew identified as a distinct group based on single-

stranded conformation polymorphism analysis of the rDNA ITS region. Mycol Res. 2005; 109:429–438.

26. Angeli D, Puopolo G, Maurhofer M, Gessler C, Pertot I. Is the mycoparasitic activity of *Ampelomyces quisqualis* biocontrol strains related to phylogeny and hydrolytic enzyme production? Biol Control 2012b; 63:348–358.

27. Kiss L, Pintye A, Kovacs GM, Jankovics T, Fontaine MC, Harvey N, Xu X, Nicot PC, Bardin M, Shykoff JA, Giraud T. Temporal isolation explains host-related genetic differentiation in a group of widespread mycoparasitic fungi. Mol Ecol. 2011; 20:1492–1507.

28. Yarwood CE. *Ampelomyces quisqualis* on clover mildew. Phytopathology 1932; 22:31. (Abstr.)

29. Szentiványi O, Kiss L. Overwintering of *Ampelomyces mycoparasites* on apple trees and other plants infected with powdery mildews. Plant Pathol. 2003; 52:737–746.

30. Sztejnberg A. Biological control of powdery mildews by *Ampelomyces quisqualis*. Phytopathology 1979; 69:1047. (Abstr.)

31. Dik AJ, Verhaar MA, Bélanger RR. Comparison of three biological control agents against cucumber powdery mildew (*Sphaerotheca fuliginea*) in semi-commercial-scale glasshouse trials. Eur J Plant Pathol. 1998; 104:413–423.

32. Jarvis WR, Slingsby K. The control of powdery mildew of greenhouse cucumber by water sprays and *Ampelomyces quisqualis*. Plant Dis Reptr. 1977; 61:728–730.

33. Shishkoff N, McGrath MT. AQ10 biofungicide combined with chemical fungicides or AddQ spray adjuvant for control of cucurbit powdery mildew in detached leaf culture. Plant Dis. 2002; 86:915–918.

34. Sundheim L. Control of cucumber powdery mildew by the hyperparasite *Ampelomyces quisquialis* and fungicides. Plant Pathol. 1982; 31:209–214.

35. Sundheim L. *Ampelomyces quisqualis*, a hyperparasitic fungus in biological control of powdery mildews on greenhouse cucumber. Acta Hortic. 1984; 156:229–236.

36. Sztejnberg A, Mazar S. Biocontrol of cucumber and carrot powdery mildew by *Ampelomyces quisqualis*. Phytopathology 1985; 75:1301–1302. (Abstr.)

37. Romero D, Rivera ME, Cazorla FM, De Vicente A, Pérez-García A. Effect of mycoparasitic fungi on the development of *Sphaerotheca fusca* in melon leaves. Mycol Res. 2003; 107:64–71.

38. Sztejnberg A, Mazar S. Studies on the hyperparasite *Ampelomyces quisqualis* and preliminary trials on biological control of powdery mildew. Phytoparasitica 1983; 11:219–220.

39. Falk SP, Gadoury DM, Pearson RC, Seem RC. Partial control of grape powdery mildew by the mycoparasite *Ampelomyces quisqualis*. Plant Dis. 1995b; 79:483–490.

40. Hofstein R, Daoust RA, Aeschlimann JP. Constrains to the development of biofungicides: the example of “AQ10”, a new product for controlling powdery mildews. Entomophaga 1996; 41:455–460.

41. Kiss L. Graminicolous powdery mildew fungi as new natural hosts of *Ampelomyces* mycoparasites. Can J Bot. 1997a; 75:680–683.

42. Mairovich G, Marder JB, Shtienberg D, Sztejnberg A. Studies of biological control of powdery mildew in cereals by the hyperparasite *Ampelomyces quisqualis*. Phytoparasitica 1996; 24:157–158. (Abstr.)

43. Pertot I, Zasso R, Amsalem L, Baldessari M, Angeli G, Elad Y. Integrating biocontrol agents in strawberry powdery mildew control strategies in high tunnel growing systems. Crop Prot. 2008; 27:622–631.

44. Pasini C, D'Aquila F, Curir P, Gullino ML. Effectiveness of antifungal compounds against rose powdery mildew (*Sphaerotheca pannosa* var. *rosae*) in glasshouses. Crop Prot. 1997; 16:251–256.

45. Gautam AK, Avasthi S. *Ampelomyces quisqualis* Ces. – a mycoparasite of *Euphorbia hirta* powdery mildew in Himachal Pradesh, India. J Phytopathol Pest Manag. 2016; 3:64–70.

46. Parratt SR, Barrès B, Penczykowski RM, Laine A-L. Local adaptation at higher trophic levels: contrasting hyperparasite-pathogen infection dynamics in the field and laboratory. Mol Ecol. 2017; 26:1964–1979.

47. Siozios S, Tosi L, Ferrarini A, Ferrari A, Tononi P, Bellin D, et al. Transcriptional reprogramming of the mycoparasitic fungus *Ampelomyces quisqualis* during the powdery mildew host-induced germination. Phytopathology 2015; 105:199–209.

48. Hijwegen T. Effect of seventeen fungicolous fungi on sporulation of cucumber powdery mildew. Neth J Plant Pathol. 1988; 94:185–190.

49. Carbó A, Torres R, Usall J, Ballesta J, Teixidó N. Biocontrol potential of *Ampelomyces quisqualis* strain CPA-9 against powdery mildew: Conidia production in liquid medium and efficacy on zucchini leaves. Sci Hortic. 2020; 267: 109337.

50. Gilardi G, Manker DC, Garibaldi A, Gullino ML. Efficacy of the biocontrol agents *Bacillus subtilis* and *Ampelomyces quisqualis* applied in combination with fungicides against powdery mildew of zucchini. JPDP 2008; 115:208–213.

51. Németh MZ, Pintye A, Horváth ÁN, Vági P, Kovács GM, Gorfer M, et al. Green fluorescent protein transformation sheds more light on a widespread mycoparasitic interaction. Phytopathology 2019; 109:1404–1416.

52. Glover BJ. Differentiation in plant epidermal cells. J Exp Bot. 2000; 51:497–505.

53. Suzuki T, Murakami T, Takizumi Y, Ishimaru H, Kudo D, Takikawa Y, et al. Trichomes: interaction sites of tomato leaves with biotrophic powdery mildew pathogens. Eur J Plant Pathol. 2018; 150:115–125.

54. Meeboon J, Hidayat I, Takamatsu S. *Pseudoidium javanicum*, a new species of powdery mildew on *Acalypha* spp. from Indonesia. Mycoscience 2013; 54:183–187.

55. Scholler M, Schmidt A, Siahaan SAS, Takamatsu S, Braun U. A taxonomic and phylogenetic study of the *Golovinomyces biocellatus* complex (Erysiphales, Ascomycota) using asexual state morphology and rDNA sequence data. Mycol Prog. 2016; 15:56.

56. Staden R, Beal KF, Bonfield JK. The Staden package, 1998. Meth Mol Biol. 2000; 132:115–130.

57. Gardes M, Bruns TD. ITS primers with enhanced specificity for basidiomycetes ‐ application to the identification of mycorrhizae and rusts. Mol Ecol. 1993; 2:113–118.

58. White TJ, Bruns T, Lee S, Taylor J. Amplification and direct sequencing of fungal ribosomal RNA genes for phylogenetics. In: Innis MA, Gelfand DH, Sninsky JJ, White TJ (eds) PCR Protocols: a guide to methods and applications, vol. 18. Academic Press, San Diego, CA; 1990. pp. 315–322.

59. Voigt K, Wöstemeyer J. Reliable amplification of actin genes facilitates deep-level phylogeny. Microbiol Res. 2000; 155:179–195.

60. Silvestro D, Michalak I. raxmlGUI: a graphical front-end for RAxML. Org Divers Evol. 2012; 12:335–337.

61. Stamatakis A. RAxML version 8: a tool for phylogenetic analysis and post-analysis of large phylogenies. Bioinformatics 2014; 30:1312–1313.

62. Huelsenbeck JP, Ronquist F. MRBAYES: Bayesian inference of phylogenetic trees. Bioinformatics 2001; 17:754–755.

63. Váczy KZ, Németh MZ, Csikós A, Kovács GM, Kiss L. *Dothiorella omnivora* isolated from grapevine with trunk disease symptoms in Hungary. Eur J Plant Pathol. 2018; 150:817–824.

64. Stöver BC, Müller KF. TreeGraph 2: Combining and visualizing evidence from different phylogenetic analyses. BMC Bioinformatics 2010; 11:7.

65. Nonomura T, Matsuda Y, Takikawa Y, Kakutani K, Toyoda H. Successional changes in powdery mildew pathogens prevailing in common and wild tomato plants rotation-cultivated in a greenhouse. Ann Rept Kansai Pl Prot. 2014; 56:17–20.

66. Nonomura T, Matsuda Y, Xu L, Kakutani K, Takikawa Y, Toyoda H. Collection of highly germinatve pseudochain conidia of *Oidium neolycopersici* from conidiophores by electrostatic attraction. Mycol Res. 2009; 113:364–372.

67. Sameshima T, Kashimoto K, Kida K, Matsuda Y, Nonomura T, Kakutani K, et al. Cytological events in tomato leaves inoculated with conidia of *Blumeria graminis* f. sp. *hordei* and *Oidium neolycopersici* KTP-01. J Gen Plant Pathol. 2004; 70:7–10.

68. Kashimoto K, Matsuda Y, Matsutani K, Sameshima T, Kakutani K, Nonomura T, et al. Morphological and molecular characterization for a Japanese isolate of tomato powdery mildew *Oidium neolycopersici* and its host range. J Gen Plant Pathol. 2003; 69:176–185.

69. Ranković B. Hyperparasites of the genus *Ampelomyces* on powdery mildew fungi in Serbia. Mycopathologia 1997; 139:157–164.

70. Vaidya S, Thakur VS. *Ampelomyces quisqualis* Ces. – a mycoparasite of apple powdery mildew in western Himalayas. Ind Phytopathol. 2005; 58:250–251.

71. Kiss L, Nakasone KK. Ribosomal DNA internal transcribed spacer sequences do not support the species status of *Ampelomyces quisqualis*, a hyperparasite of powdery mildew fungi. Curr Genet. 1998; 33:362–367.

72. Gu YH, Ko WH. Water agarose medium for studying factors affecting germination of conidia of *Ampelomyces quisqualis*. Mycol Res. 1997; 101:422–424.

73. De Bary A. *Eurotium*, *Erysiphe*, *Cicinnobolus*: Nebst Bemerkungen über die Geschlechtsorgane der Ascomyceten. In: Beitrage zur Morphologie und Physiologie der Pilze, De Bary A, Woronin M (eds), Verlag von C. Winter, Frankfurt AM, Germany; 1870. pp.1–95.

74. Philipp W-D, Crüger G. Parasitismus von *Ampelomyces quisqualis* auf echten mehltaupilzen an gurken und anderen gemüsearten. Z Pflanzenkrankh Pflanzenschutz 1979; 86:129–142.

75. Speer EO (1978) *Ampelomyces cesati* (Fungi, Sphaeropsidales). Taxon 27:549–562.

76. Kiss L, Pintye A, Zséli G, Jankovics T, Szentiványi O, Hafez YM, et al. Microcyclic conidiogenesis in powdery mildews and its association with intracellular parasitism by *Ampelomyces*. Eur J Plant Pathol. 2010; 126:445-451.

77. Sundheim L, Krekling T. Host-parasite relationships of the hyperparasite *Ampelomyces quisqualis* and its powdery mildew host *Sphaerotheca fuliginea*. Phytopath Z. 1982; 104:202–210.

78. Zeng LF, Jiang YC. An investigation of parasitic fungi on *Erysiphe* spp. RoPP 1986; 65:419.

79. Rotem Y, Yarden O, Sztejnberg A. The mycoparasite *Ampelomyces quisqualis* expresses *exgA* encoding and *exo*-β-1,3-glucanase in culture and during mycoparasitism. Phytopathology 1999; 89:631–638.

80. Hashioka Y, Nakai Y. Ultrastructure of pycnidial development and mycoparasitism of *Ampelomyces quisqualis* parasitic on Erysiphales. Trans Mycol Soc Japan 1980; 21:329–338.

Legends of Figures

Fig 1. Phylogenetic tree with the highest likelihood value resulting from the maximum likelihood (ML) analysis of ITS and *ACT* sequences of 129 *Ampelomyces* strains, including 26 isolated in this study (written in bold) in Japan. The tree is rooted to *Phoma herbarum* strain CBS56763 based on an earlier study [17]. Bootstrap values calculated from 1000 replicates in ML analysis are written as percentages (values below 70% and in subclades are not shown). These are followed by posterior probabilities resulting from Bayesian analysis (no values are shown below 0.9 and in subclades), separated by slashes. Bar indicates 0.02 expected changes per site per branch. Additional data on *Ampelomyces* strains (place and data of collection, host fungal species) and GenBank accession numbers of sequences included in the analyses are listed in earlier studies [17,22].

Fig 2. Digital (A and B) and light micrographs (C) of *Ampelomyces* strain Xs-q on tomato powdery mildew KTP-03 mycelia. A and B, mature (Py1) and immature pycnidia (Py2) formed onto KTP-03 mycelia. B, Pycnidium (Py) produced in KTP-03 conidiophores (Cp). C, Spores appeared from a pycnidium after ~~treatment of~~ a drop of distilled water (10 µm). Micrographs were taken at 14 days after spray inoculation of spores onto 10-day-old KTP-03 mycelia. Bars represent 50 µm (A) and 30 µm (B and C). Hp, Hypha of tomato powdery mildew KTP-03.

Fig 3. Digital (A and B) and light micrographs (C to F) of *Ampelomyces* strain Xs-q in tomato powdery mildew KTP-03. A, KTP-03 hyphae infected tomato type I trichome cells. B, Xs-q hyphae invaded into KTP-03 conidium. Arrow shows invasion site of Xs-q hypha. C, Xs-q hyphae grown into

KTP-03 hyphae and primary conidiophore. D, Xs-q hyphae grown and pycnidium produced in a KTP-03 conidiophore. E, Xs-q pycnidium produced in a KTP-03 conidiophore and abundant spores appeared from the pycnidium. F, Xs-q pycnidia produced in KTP-03 conidiophores formed on tomato type I trichome cell. Bars represent 60 μm (A and F) and 20 μm (B to E). Ahp, Xs-q hypha; Co, KTP-03 conidium; Cp, KTP-03 conidiophore; Hp, KTP-03 hypha; Py, Xs-q pycnidium; Sp, Xs-q spore; Tc, Tomato type I trichome cell.

Fig 4. Infection process of *Ampelomyces* strain Xs-q in tomato powdery mildew KTP-03. KTP-03 vigorously grew on tomato type I trichome cells. Digital micrographs were taken at 0 (A), 2 (B), 3 (C), 5 (D), 6 (E) and 7 days (F) after inoculation of a pycnidium onto tomato leaf in the vicinity of the trichome cells. KTP-03 conidiophores (Cp) and hyphae (arrowed) atrophied after invasion of Xs-q hyphae (Ahp) into KTP-03 hyphae. Bars represent 20 μm.

Fig 5. Pycnidial formation processes of *Ampelomyces* strain Xs-q in KTP-03. A KTP-03 mycelium was formed on tomato leaf at 10 days after inoculation of a KTP-03 conidium with micromanipulation technique. Digital micrographs were taken at 0 (A), 5 (B), 7 (C), 10 (D) and 12 days (E) after spray inoculation of Xs-q spores onto the 10-day-old KTP-03 mycelium. KTP-03 conidiophores (Cp), formed near the trichome cell (Tc), completely atrophied after invasion of Xs-q hyphae (Ahp) into KTP-03 hyphae (arrows). Xs-q pycnidia (Py) were successfully produced in KTP-03 conidiophores. Bars represent 20 μm.

Figure 1

Click here to access/download;Figure;Fig. 1.tif

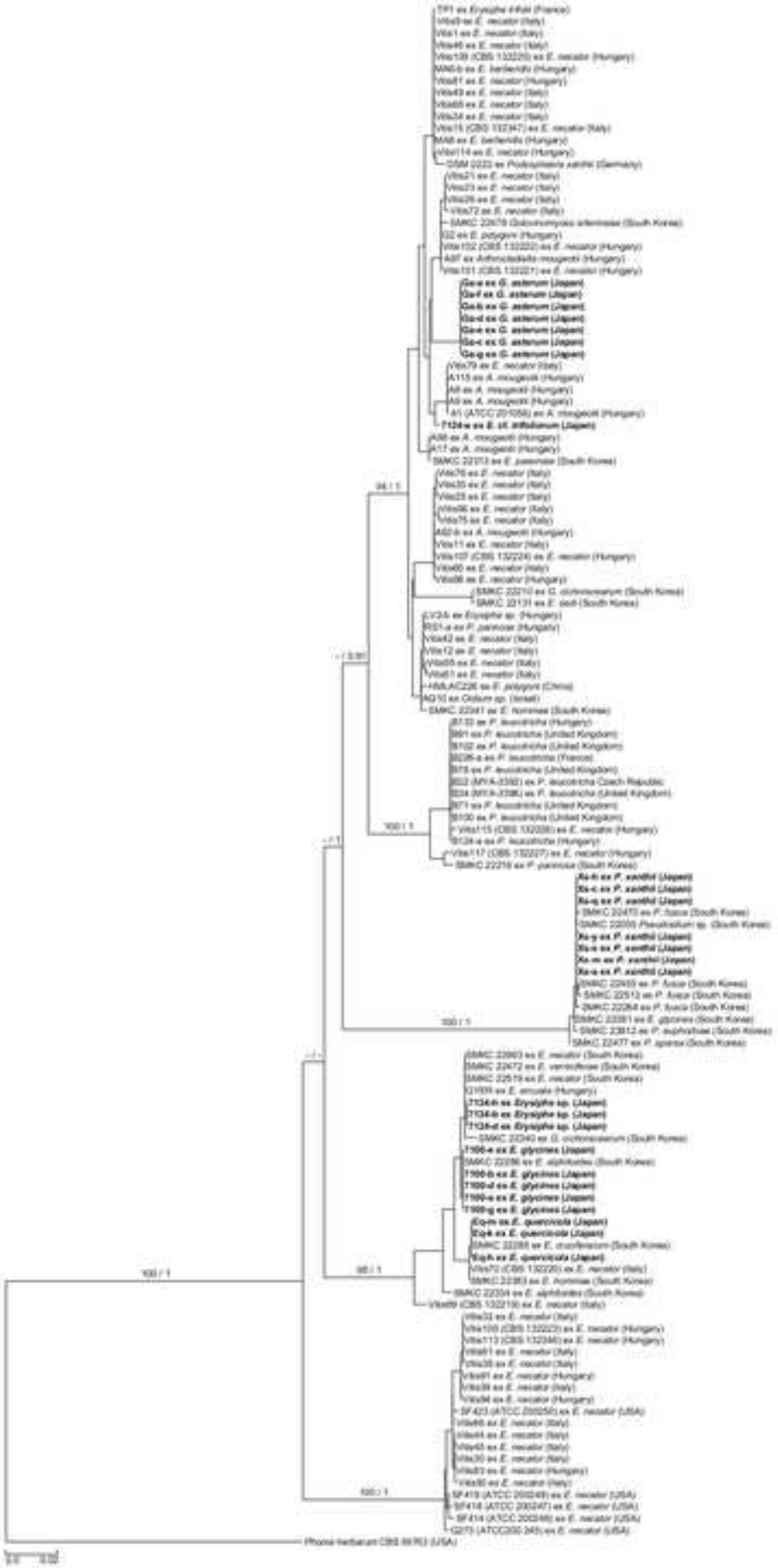

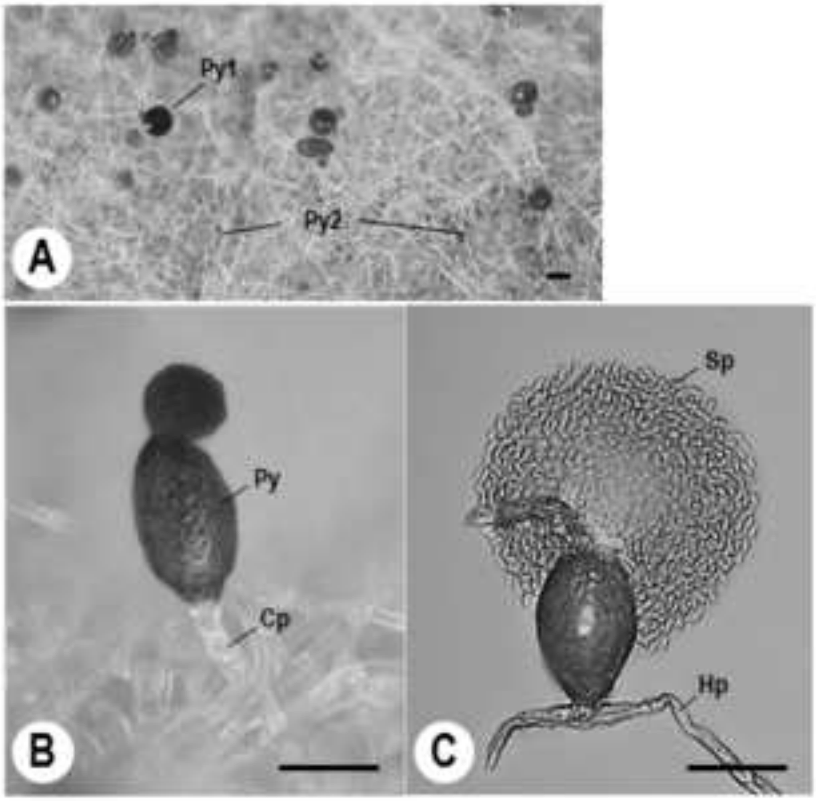

Fig. 2

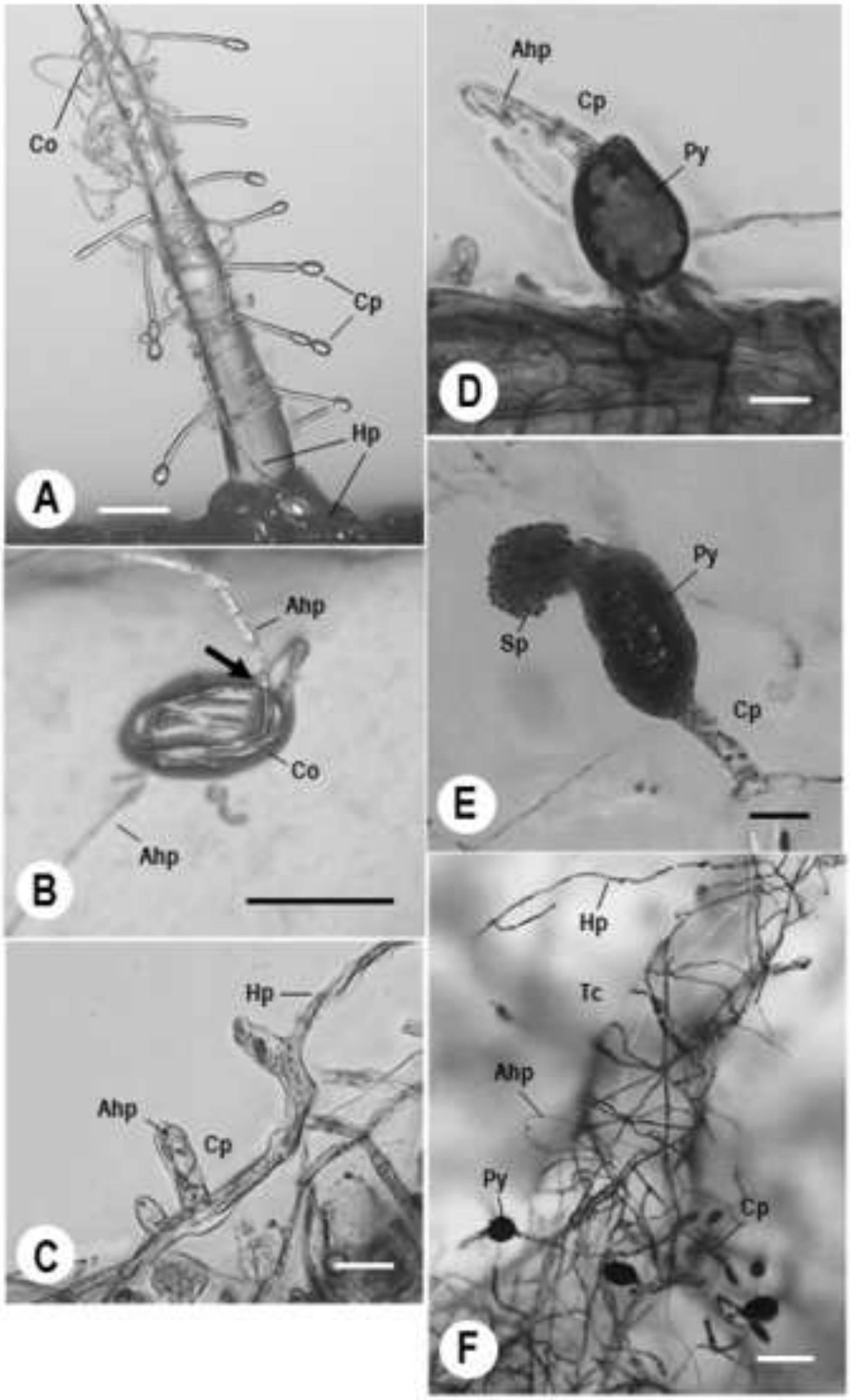
Co
Cp
Hp
A
Ahp
Co
Ahp
B
Hp
Ahp
Cp
C
Ahp
Cp
Py
D
Py
Sp
Cp
E
Hp
Tc
Ahp
Py
Cp
F

Fig. 3

 Click here to access/download;Figure;Fig. 4.tif

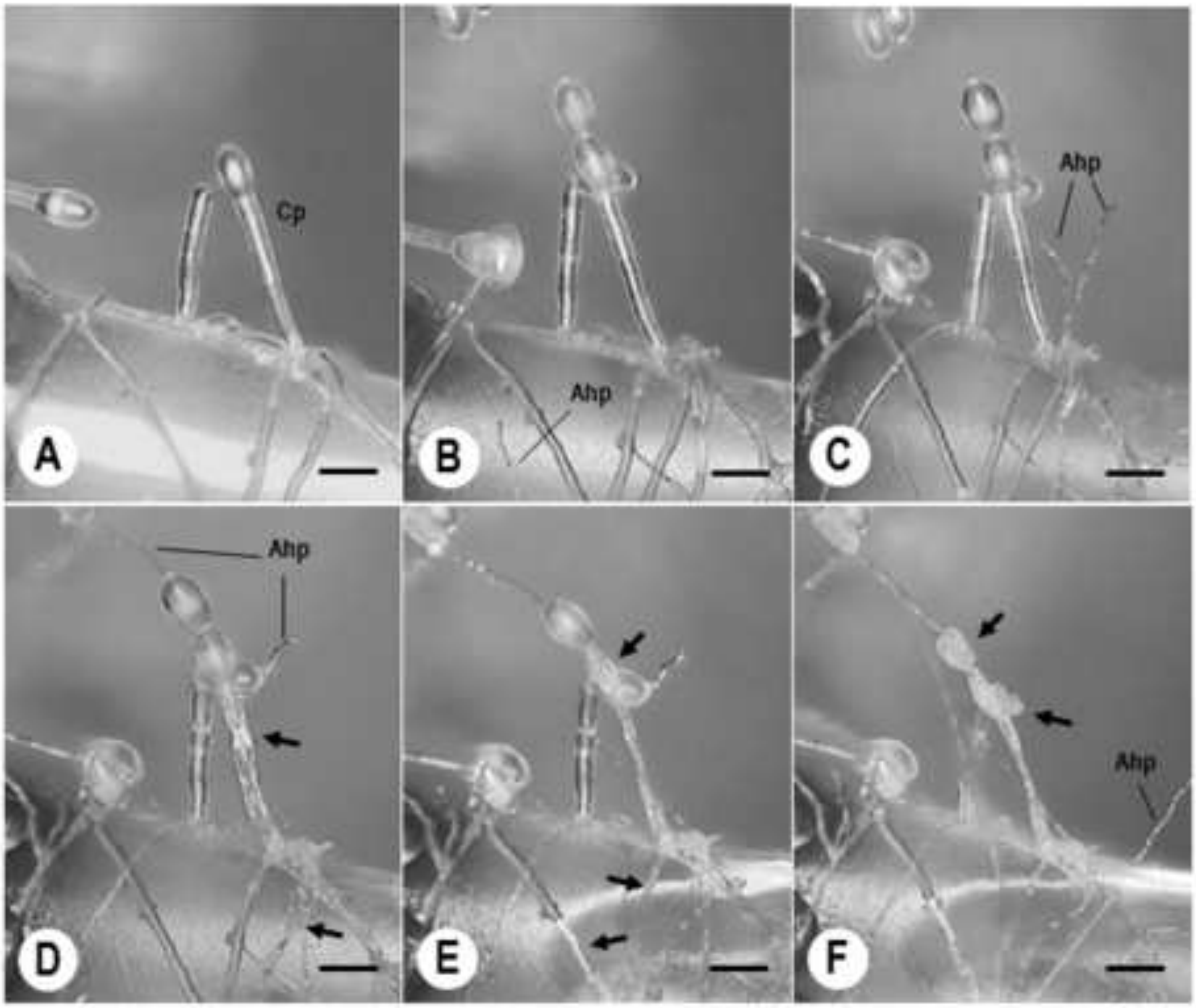

Fig. 4

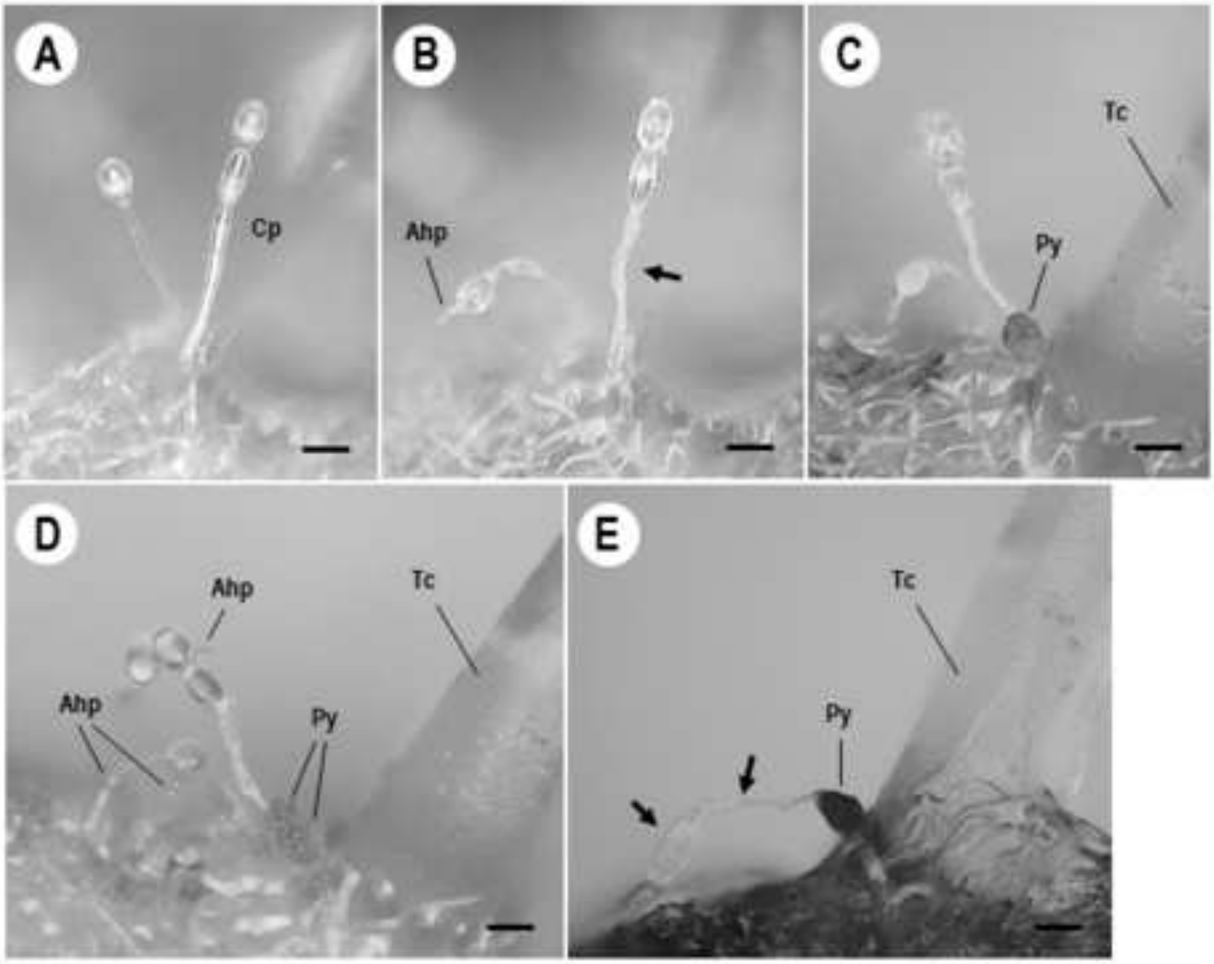

Fig. 5
